# Modeling analysis of secondary inorganic aerosols over China: pollution characteristics, and meteorological and dust impacts

**DOI:** 10.1038/srep35992

**Published:** 2016-10-26

**Authors:** Xiao Fu, Shuxiao Wang, Xing Chang, Siyi Cai, Jia Xing, Jiming Hao

**Affiliations:** 1 State Key Joint Laboratory of Environment Simulation and Pollution Control, School of Environment, Tsinghua University, Beijing 100084, China; 2 Department of Civil and Environmental Engineering, Hong Kong Polytechnic University, Hong Kong, China; 3 State Environmental Protection Key Laboratory of Sources and Control of Air Pollution Complex, Beijing 100084, China; 4 Collaborative Innovation Center for Regional Environmental Quality, Tsinghua University, Beijing 100084, China

## Abstract

Secondary inorganic aerosols (SIA) are the predominant components of fine particulate matter (PM_2.5_) and have significant impacts on air quality, human health, and climate change. In this study, the Community Multiscale Air Quality modeling system (CMAQ) was modified to incorporate SO_2_ heterogeneous reactions on the surface of dust particles. The revised model was then used to simulate the spatiotemporal characteristics of SIA over China and analyze the impacts of meteorological factors and dust on SIA formation. Including the effects of dust improved model performance for the simulation of SIA concentrations, particularly for sulfate. The simulated annual SIA concentration in China was approximately 10.1 μg/m^3^ on domain average, with strong seasonal variation: highest in winter and lowest in summer. High SIA concentrations were concentrated in developed regions with high precursor emissions, such as the North China Plain, Yangtze River Delta, Sichuan Basin, and Pearl River Delta. Strong correlations between meteorological factors and SIA pollution levels suggested that heterogeneous reactions under high humidity played an important role on SIA formation, particularly during severe haze pollution periods. Acting as surfaces for heterogeneous reactions, dust particles significantly affected sulfate formation, suggesting the importance of reducing dust emissions for controlling SIA and PM_2.5_ pollution.

Secondary inorganic aerosols (SIA; i.e., sulfate, nitrate, and ammonium) are the predominant components of fine particles (PM_2.5_) in China, making up about 30–40% of total PM_2.5_ mass annually^1^,^2^, and even over 50% of PM_2.5_ mass during some heavy pollution episodes^3^. Previous studies have demonstrated that SIA has considerable impacts on human health, visibility, and climate change^4^,^5^,^6^,^7^.

Numerical modeling is a useful method for analyzing the pollution characteristics and crucial processes involved in SIA formation. SIA is generated mainly through the oxidation and neutralization of sulfur dioxide (SO_2_), nitrogen oxides (NO_X_), and ammonia (NH_3_). Chemical transport models consider traditional oxidation paths, such as SO_2_ oxidization by the hydroxyl radical (OH) in the gas phase, or by dissolved hydrogen peroxide (H_2_O_2_), ozone (O_3_), oxygen (O_2_), and organic peroxides in the aqueous phase. The formation paths of nitric acid (HNO_3_) include the gas-phase oxidation of OH during the day and the hydrolysis of nitrogen pentoxide (N_2_O_5_) at night. However, numerous studies have demonstrated that chemical transport models cannot simulate SIA well, especially underestimating sulfate concentrations significantly^8^,^9^,^10^,^11^. This bias could probably be attributed to the lack of some path in the transition of SO_2_ to sulfate.

The results of field observations and laboratory studies suggest that dust particles can enhance sulfate concentration. First, dust particles are rich in transition metals such as iron (Fe) and manganese (Mn), which can catalyze the oxidation of dissolved SO_2_ by O_2_^12^. Using isotopic analysis, Harris *et al*. identified this pathway as the dominant in-cloud oxidation pathway for sulfate formation during their observation period in Germany^13^. In most current modeling studies, the sulfate generated by the catalyzed oxidation of SO_2_ was not considered because of the absence of this mechanism in the model used or the lack of emissions. Furthermore, numerous laboratory studies have confirmed the existence of heterogeneous reactions of SO_2_ on the surface of dust particles^14^,^15^,^16^. However, this pathway is not considered in most chemical transport models.

In this study, we estimated the emissions of dust particles and chemical components. To improve the model’s capability to reproduce SIA concentrations, we incorporated the heterogeneous reactions of SO_2_ on the surface of dust particles into the Community Multiscale Air Quality (CMAQ) modeling system, which is a widely used regional chemical transport model^17^,^18^,^19^. Pollution characteristics and the impacts of meteorological parameters and dust particles on SIA were investigated through full-year simulations over China by using the revised CMAQ models. The results of this study increase our understanding of the characteristics of SIA pollution in China and provide new insights on air pollution control strategies.

## Results

### Simulation of ambient SIA

We considered the impacts of dust particles to obtain better model performance in the SIA simulation, specifically for sulfate concentrations. First, we estimated the emissions of dust aerosols, including from erodible lands, roads, and construction activities. The dust emissions from erodible lands were calculated using the in-line windblown dust model in the CMAQ. The threshold friction velocity for loose, fine-grained soil was revised according to Chinese monitoring data to increase the capacity of the model to simulate windblown dust in China^20^. Information on erodible lands, including shrub land, shrub grass, and barren land, was derived from Moderate Resolution Imaging Spectroradiometer (MODIS) data. The emission factor approach was used to estimate the fugitive dust emissions from roads and construction activities (see Supplementary Section 1). In addition to the total dust emissions, we estimated the emissions of the key dust components that influence sulfate generation, including Ca^2+^, Fe (III), and Mn (II) (see Supplementary Section 1). Ca^2+^ increases the pH of cloud water, which affects the rate of aqueous-phase oxidation of S(IV). Fe(III) and Mn(II) catalyze the aqueous oxidation of S(IV) by O_2_. Based on the estimated dust emissions, the heterogeneous reactions of SO_2_ on the surface of dust particles were incorporated into the CMAQ modeling system (see Supplementary Section 2).

**Figure 1:**
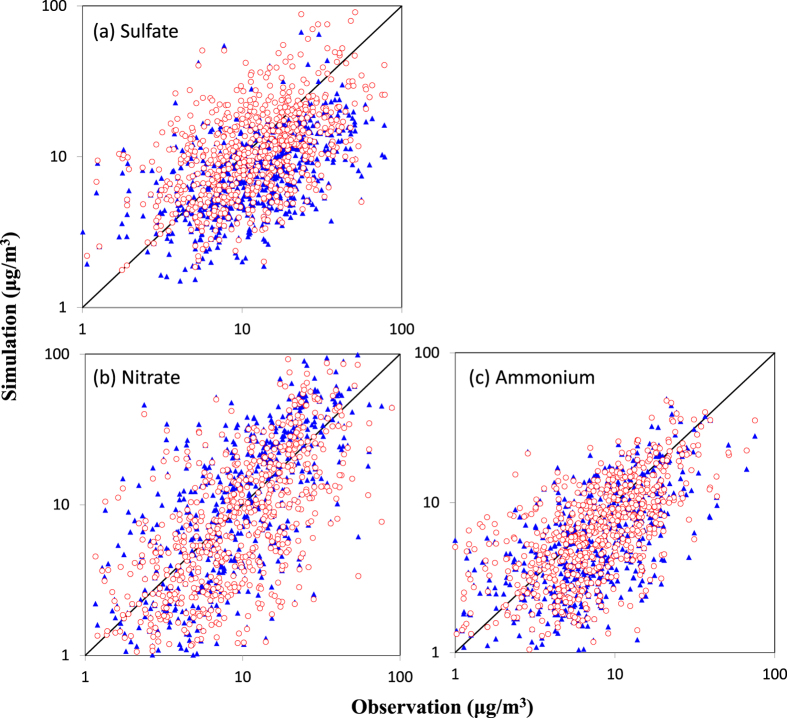
Comparison of the simulated and observed daily concentrations for sulfate, nitrate and ammonium. Blue triangles are results for Simulation I, and red circles are results for Simulation II.

Two parallel experiments were conducted in a 36 km by 36 km simulation domain covering the whole China (see Supplementary Fig. 1). The default CMAQ modeling system was used in Simulation I, whereas sulfate enhancement by dust was considered in Simulation II. Daily observations at eight monitoring sites were used to evaluate the model performance for SIA simulation (see Supplementary Fig. 1 and Supplementary Table 5). The default CMAQ modeling system significantly underestimated sulfate concentrations, with a normalized mean bias (NMB) of −41.3% (see Supplementary Table 6). With the inclusion of dust impacts, the revised CMAQ model reduced the NMB to −12.3%, and increased the R (correlated coefficient) value from 0.4 to 0.5 (Fig. 1). NH_3_ partitioning with SO_4_^2−^ and NO_3_^−^ generates SIA, and (NH_4_)_2_SO_4_ is the preferential species. Because of the increase in simulated sulfate concentration, the revised simulation reduced the overestimation of nitrate concentrations and the underestimation of ammonium concentrations. The NMB was reduced from 24.2% to −3.3% for nitrate and from −21.6% to −16.5% for ammonium.

### Spatial and seasonal patterns of SIA over China

**Figure 2:**
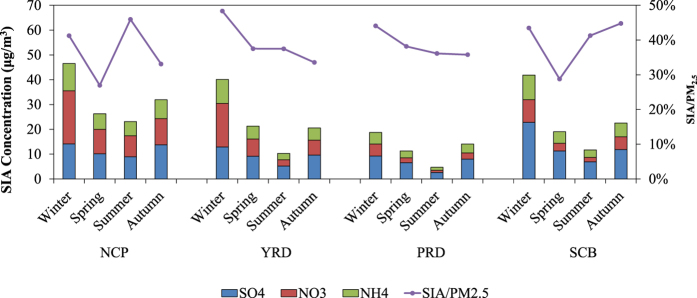
Simulated SIA concentrations and the ratios of SIA/PM2.5 in different season for the NCP, YRD, PRD and SCB region.

The concentrations of SIAs are shown to be highest in winter, followed by autumn and spring, and lowest in summer (see Supplementary Fig. 3). This seasonal pattern was affected by precursor emissions, wet deposition, and diffusion conditions. In winter, SO_2_ and NO_X_ emissions were highest due to more coal consumption associated with heating activities in north China. Additionally, the planetary boundary layer (PBL) height and precipitation were also lowest in winter, creating favorable conditions for SIA accumulation. Moreover, the low temperatures during winter were favorable for nitrate to stay aerosol phase. High SIA concentrations were concentrated in some developed regions with high precursor emissions, such as the North China Plain (NCP), Yangtze River Delta (YRD), Sichuan Basin (SCB), and Pearl River Delta (PRD). The total SIA concentrations in these regions were 32.0, 23.1, 23.8 and 12.2 μg/m^3^, accounting for 36%, 40%, 40% and 39% of PM_2.5_ concentrations, respectively (Fig. 2). NO_3_^−^ and NH_4_^+^ concentrations were highest in the NCP region, whereas SO_4_^2−^ concentrations were highest in the SCB region, agreeing with the spatial pattern of the precursor emissions. In winter, SIA pollution was most severe and the SIA proportions in PM_2.5_ were also high, at >40% for all regions. The NCP and SCB regions exhibited the lowest SIA proportions in spring because of largest contributions from windblown dust. For the YRD and PRD regions, the SIA proportions were comparatively lower in autumn, which was consistent with the observations in these regions^21^,^22^.

### Meteorological impacts on SIA pollution levels

**Figure 3:**
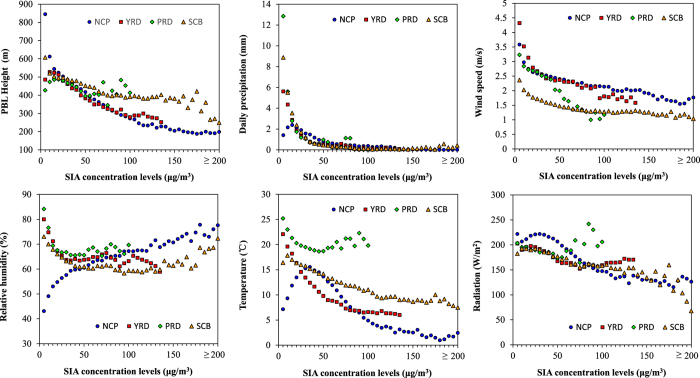
The correlations between meteorological factors and SIA pollution levels.

We analyzed meteorological characteristics under different SIA pollution levels in the four aforementioned regions to increase our understanding of the characteristics and causes of SIA pollution. Six meteorological factors were considered. PBL height, precipitation, and wind speed were considered because they affect the physical processes of SIA removal and dilution, such as diffusion and deposition. Additionally, relative humidity, temperature, and solar radiation were considered because they affect the chemical processes of SIA generation, such as heterogeneous oxidation, thermodynamic reactions, and photochemical oxidation. As shown in Fig. 3, “X-coordinate” refers to the 21 SIA concentration levels from 0 to >200 μg/m^3^ with an interval of 5 μg/m^3^. “Y-coordinate” refers to the mean value of the meteorological factor in all the grids where the daily SIA concentration was within the specific SIA concentration interval. Meteorological characteristics in each region were not the same, because of difference in latitude and topography. In the NCP region, decreasing PBL height and wind speed aggravated SIA accumulation more than precipitation did. Additionally, the SIA concentration presented a significant positive correlation with relative humidity, which was attributed to the enhanced heterogeneous generation under high relative humidity and stable meteorological conditions^3^,^23^. As expressed in the Supplementary Equation 4, the uptake coefficient of SO_2_ heterogeneous reactions increased rapidly with increasing relative humidity. The similar positive correlation existed in the SCB region under heavy SIA pollution level (>150 μg/m^3^). At the same time, a negative correlation existed between SIA concentration and radiation in the NCP and SCB regions, indicating that heterogeneous oxidation under high relative humidity was more critical for SIA generation than photochemical oxidation was, particularly under severe SIA pollution. But, in the PRD region, photochemical oxidation would be more important. First, solar radiation in the PRD was stronger than that in the NCP and SCB because of the region’s lower latitude. These factors resulted in stronger photochemical oxidation in the PRD compared to the NCP and SCB regions. Dust concentrations in the PRD were also comparatively lower, which led to lower heterogeneous oxidation. Additionally, for the PRD region, among three meteorological factors affecting physical processes, wind speed decreased significantly under SIA pollution condition, but PBL height and precipitation remained relatively stable. This indicated that low wind speed was a critical factor of SIA pollution in this region. In all regions except the PRD, a distinct negative correlation existed between SIA concentrations and temperature. Lower temperature was favorable for NH_4_NO_3_ to keep stable.

### Sulfate enhancement by dust particles

**Figure 4:**
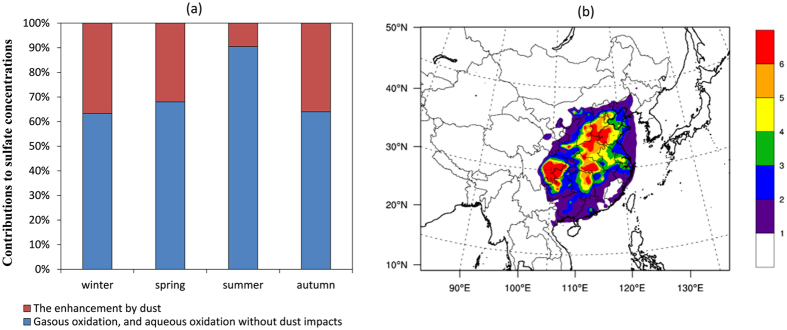
(**a**) Seasonal contributions of different pathways to sulfate production in China; (**b**) Spatial distribution of the increased sulfate concentrations (μg/m^3^) caused by dust impacts. This figure is produced using the NCAR Command Language (Version 6.2.1) [Software]. (2014). Boulder, Colorado: UCAR/NCAR/CISL/TDD. http://dx.doi.org/10.5065/D6WD3XH5, and Microsoft office 2013 (https://www.microsoft.com/).

By comparing the simulation results with and without dust impacts, we quantified the sulfate enhancement by dust impacts. The aggregate sulfate concentration was increased by 1.4 μg/m^3^, averagely. As shown in Fig. 4a, the seasonal sulfate enhancement percentage was highest in winter (37%) and lowest in summer (10%). The main reason was that low precipitation and stable atmospheric structure in winter were favorable for the accumulation of dust particles and thus provided more reactive interfaces for the heterogeneous generation of SO_2_. Additionally, the concentrations of oxidative gases, such as OH, O_3,_ and H_2_O_2_, were low in winter because of less solar radiation. This weakened the importance of gaseous and aqueous oxidation by OH, O_3_, and H_2_O_2_. In contrast, in summer, the dust particle concentrations were low, but the oxidant concentrations were high. Dust particles caused high sulfate enhancement in the SCB and NCP regions, with averages of 5.3 and 4.5 μg/m^3^, respectively (Fig. 4b). These two regions were greatly affected by high SO_2_ and dust particle pollution. The simulated regional annual average SO_2_ concentrations of the SCB and NCP regions were 24.1 and 30.1 μg/m^3^, and their annual average fine dust particle concentrations were 17.3 and 25.8 μg/m^3^, respectively. These concentrations were considerably denser than those in other parts of China.

The enhancement of sulfate formation by dust particles was also important in some heavy pollution episodes. For instance, a severe winter haze occurred over the North China in January, 2013, when the highest PM_2.5_ concentration could be above 500 μg/m^3^ and the sulfate concentration presented a rapid growth when the haze occurred^3^,^24^. He *et al*.^24^ discovered that the increased sulfate proportion on haze days was accompanied by an increase in the mineral proportion, indicating the importance of dust particles in sulfate generation. We analyzed the impacts of dust during the heaviest pollution episode (January 9–17, 2013). As shown in Supplementary Fig. 4, an average sulfate concentration of 51.6 μg/m^3^ for the whole episode was measured at a site in Tsinghua University, Beijing (40.0°N, 116.3°E)^25^. The corresponding simulated average sulfate concentration for the entire episode increased from 18.4 to 30.6 μg/m^3^. The daily enhancement for sulfate concentration was highest on January 12, at approximately 31 μg/m^3^. These results suggest the importance of reducing dust emissions for controlling SIA and PM_2.5_ pollution.

## Discussion

Several uncertainties and limitations still exist in this study. First, the uptake coefficients of the heterogeneous reactions of SO_2_ on dust particles surface and the RH-dependent parameterization were identified according to limited literature. Because of the lack of quantitative laboratory results in the current literature, we neglected the impacts of temperature, dust particle compositions, surface conditions, and NO_X_ promotion^24^,^26^. Additional studies are required to quantify the uptake coefficients of SO_2_ on dust particle surfaces more accurately. Second, the model system used in this study did not include the aerosol direct effects, which could enhanced SIA concentrations^27^ and improved the underestimation of sulfate concentration during episodes of severe pollution. Furthermore, the estimation of fugitive dust emissions involved high uncertainty because of limited local measurements for emission factors and the lack of accurate location information. More accurate estimates of SIA precursors and dust emissions are required for simulating SIA concentrations and dust impacts better.

## Methods

We incorporated the SO_2_ heterogeneous reactions on the dust surface to the CMAQ model version 5.0.2. The uptake of the heterogeneous reaction was parameterized by a pseudo-first-order rate constant. We considered the impacts of relative humidity on the uptake coefficient^28^ and the uptake coefficient under the dry condition was identified as to be 6 × 10^−5^ referring to previous studies on the interaction between SO_2_ and dust particles^14^,^15^,^16^,^29^ (see Supplementary Section 2). Additionally, we estimated the emissions of dust aerosols, including from erodible lands, road and construction activities. The dust emissions from erodible lands were calculated by the revised in-line windblown dust model in the CMAQ. The fugitive dust emissions from road and construction activities were estimated using an emission factor approach (see Supplementary Section 1). We also estimated the emissions of the key components of dust affecting sulfate generation, including Ca^2+^, Fe (III) and Mn (II) (see Supplementary Section 1).

We applied both the revised and default CMAQ models over a simulation domain covering the whole China, with a grid resolution of 36 km × 36 km. It is based on a Lambert projection with the two true latitudes of 25°N and 40°N and an origin of 34°N, 110°E. The coordinates of the bottom left corner were (x = −3114 km, y = −2448 km). Twenty-three vertical layers were used from the surface to the tropopause. The gas-phase chemistry and aerosol modules chosen in the CMAQ model were the CB05 chemical mechanism and the AERO6 model. The meteorological fields for the CMAQ simulations were generated using the Weather Research and Forecasting (WRF) model, version 3.5. Because considerable changes and developments have occurred in China in the past 20 years, the default land-use data was outdated. Therefore, we updated the land-use data in the WRF using MODIS data. The other WRF data sources and major physics options were same as described in our previous paper^20^. The model performance for the meteorological prediction was reasonably acceptable (see Supplementary Table 3). Except the dust emissions, the emissions of other pollutants were estimated using the method described in our previous paper^30^. The activity data, and technology distribution for each sector were updated. The emissions of NH_3_ from the fertilizer application were calculated online using the bi-directional CMAQ model^31^. Compared with previous studies, this method considered more influencing factors, such as meteorological fields, soil and fertilizer application, and provided improved spatial and temporal resolution. The biogenic emissions were calculated by the Model of Emissions of Gases and Aerosols from Nature (MEGAN)^32^.

The model simulation period is the whole year of 2013. We also selected additional simulation periods of 2011 based on availability of observational data. The model evaluation was described in detail in supplementary section 3. We defined four key metropolitan regions for region-specific analysis, as shown in Supplementary Fig. 1.

## Additional Information

**How to cite this article**: Fu, X. *et al*. Modeling analysis of secondary inorganic aerosols over China: pollution characteristics, and meteorological and dust impacts. *Sci. Rep.* **6**, 35992; doi: 10.1038/srep35992 (2016).

**Publisher’s note:** Springer Nature remains neutral with regard to jurisdictional claims in published maps and institutional affiliations.

## Supplementary Material

Supplementary Information
