# Supplementary material for: Modeling analysis of secondary inorganic aerosols over China: pollution characteristics, and meteorological and dust impacts
Source: Sci Rep. 2016 Oct 26;6:35992. doi: 10.1038/srep35992 (PMC5080613; doi:10.1038/srep35992)
Supplement: Supplementary Information [file srep35992-s1.pdf]

Supplementary material for the paper entitled:

Modeling analysis of secondary inorganic aerosols over China: pollution characteristics, and meteorological and dust impacts

Xiao Fu [1, 2], Shuxiao Wang [1, 3], Xing Chang [1], Siyi Cai [1], Jia Xing [1], Jiming Hao [1, 4]

[1] State Key Joint Laboratory of Environment Simulation and Pollution Control, School of Environment, Tsinghua University, Beijing 100084, China

[2] Department of Civil and Environmental Engineering, Hong Kong Polytechnic University, Hong Kong, China

[3] State Environmental Protection Key Laboratory of Sources and Control of Air Pollution Complex, Beijing 100084, China

[4] Collaborative Innovation Center for Regional Environmental Quality, Tsinghua University, Beijing 100084, China

Correspondence to: S.X.Wang (shxwang@tsinghua.edu.cn)

1. Emission inventory

Dust emission. In this study, we estimated the emissions of fugitive dust, including from erodible lands, road and construction activities.

The dust emissions from erodible lands were calculated by the in-line windblown dust model in the CMAQ. The threshold friction velocity for loose, fine-grained soil was revised based on Chinese monitoring data, so that the model can reflect the windblown dust in China better[1]. The erodible lands included shrub land, shrub grass and barren land, which were extracted from the MODIS data.

An emission factor approach was used to estimate the fugitive dust emissions from road and construction activities. For the fugitive dust from road, the emissions were calculated as follows:

$$E_{road} = EF_{road} \times \sum_{i} (P_i \times VKT_i) \tag{1}$$

Where i represents vehicle types, including heavy bus (HB), medium bus (MB), light bus (LB), mini bus (MINIB), heavy truck (HT), medium truck (MT), light truck (LT) and mini truck (MINIT). P_i is the vehicle populations for vehicle type i, which were extracted from the statistical yearbooks. VKT_i is the vehicle kilometers of travel for vehicle type i, which were referred to the study of Zheng et al.[2] EF_{road} is emission factors, which were set as 1.93 and 0.33g/VKT for PM_{10} and $PM_{2.5}$, respectively, referring to the Chinese local measurements[3-5].

For the fugitive dust from construction activities, the emissions were calculated as follows:

$$E_{construction} = EF_{construction} \times (A \times r \times T) \tag{2}$$

Where A is construction area, which was extracted from the statistical yearbooks. r is the volume ratio, which was set as 2.68. T is construction time, which was set as 163 days per year. $EF_{construction}$ is emission factors, which were set as 0.128 and 0.026 kg/(m^2·month) for PM_{10} and $PM_{2.5}$, respectively. These values were chosen based on the Chinese local study[6-10].

In 2013, the emissions of PM_{10} and $PM_{2.5}$ for fugitive dust from road were 5242 kt and 904 kt. The emissions of PM_{10} and $PM_{2.5}$ for fugitive dust from construction activities were 2895 kt and 579 kt.

Key components of dust affecting sulfate generation: Ca^{2+}, Fe(III) and Mn(II). In addition to the total emissions of dust, we also estimated the emissions of the key components

of dust affecting sulfate generation, including Ca^{2+}, Fe (III) and Mn (II). Ca^{2+} could increase the pH value of cloud water, affecting the rate of aqueous-phase oxidation of S(IV). Fe(III) and Mn(II) could catalyze the S(IV) aqueous oxidation by O_2.

First, we collected the Chinese local measurements data for element Ca, Fe and Mn in $PM_{2.5}$ from fugitive dust[11-17], as shown in **Supplementary Table 1**. The average values of these studies were used. For water-soluble Ca^{2+}, the values of Ca^{2+}/Ca were set as 30%, 50% and 14% for fugitive dust from desert, road and construction activities, respectively, referring to the Chinese local studies[17-21]. Similar with Alexander et al.[22] and Huang et al.[23], the mass fractions of soluble Fe and Mn were assumed as 10% and 50%, respectively. Mn existed mainly as Mn(II) in cloud/fog water, and Mn(II) was assumed to be 100% of the dissolved Mn. 10% of the dissolved Fe was assumed to be Fe(III) during the day and 90% at night because iron cycles diurnally[24].

Supplementary Table 1.The mass fractions of element Ca, Fe and Mn in $PM_{2.5}$ from fugitive dust

Source	Fe	Mn	Ca	Measurement place	Reference
Desert	5.63	0.11	2.99	Taklimakan Desert	Zhang et al. (2014)[15]
	5.77	0.13	3.90	Xinjiang Gobi	
	5.81	0.14	2.48	Anxinan Gobi	
	2.93	0.08	3.77	Ulan Buh Desert	
	2.13	0.04	1.44	Central Inner Mongolia Desert	
	2.88	0.07	4.14	Erenhot Gobi	
Road	4.99		5.71	Zhangzhou	Zheng et al. (2013)[17]
	4.29		6.45	Quanzhou	
	4.69		6.01	Putian	
	6.56	0.10	3.76	Hangzhou	Bao et al. (2010)[11]
	2.76	0.09	7.78	Beijing	Ma et al. (2015)[14]
	6.16	0.11	7.75	Hong Kong	Ho et al. (2003)[12]
Construction activities	2.42	0.05	20.51	Beijing	Hua et al. (2006)[13]
	3.67	0.11	20.48	Tianjin	Zhao (2008)[16]

3.37	0.10	23.63	Hangzhou	Bao et al. (2010)[11]

Other pollutants. We also estimated the emissions of other pollutants for China, including SO_2, NO_X, PM_{10}, $PM_{2.5}$, NMVOC, and NH_3. The method used to develop the emission inventory was described in our previous paper[25], in which the emission inventory for 2010 was developed and verified. The activity data, and technology distribution for each sector were updated. The emissions of NH_3 from the fertilizer application were calculated online using the bi-directional CMAQ model[26]. Compared with previous researches, this method considers more influencing factors, such as meteorological fields, soil and fertilizer application, and provides improved spatial and temporal resolution. The biogenic emissions were calculated by the Model of Emissions of Gases and Aerosols from Nature (MEGAN)[27].

2. Heterogeneous reaction of SO_2 on dust surface and its implementation into CMAQ

In this study, the heterogeneous reaction of SO_2 on dust surface was incorporated into the original CMAQ model. The uptake of this reaction is commonly parameterized by a pseudo-first-order rate constant[28], which is as follows:

$$k_g = \left(\frac{r_p}{D_g} + \frac{4}{v_g \gamma_g} \right)^{-1} A_p \qquad (3)$$

Where r_p and A_p are the radius and surface area density of dust particles, D_g is the gas-phase molecular diffusion coefficient of SO_2, v_g is the mean molecular velocity of SO_2 and γ_g is the uptake coefficient. The parameters r_p, A_p, D_g and v_g were calculated in the CMAQ model, and the estimation of dust emission has been described in section 1 in detail.

The studies based on laboratory experiments and field observations all showed that the values of γ_g increased rapidly with the growing of RH, especially when RH was higher than 50%[29-31]. In this study, we referred to the function in the study of Sun et al. (2013)[31] to represent the RH-dependence of γ_g, which is as follows:

$$\gamma_g = \gamma_{RH=0} \times \frac{\left[0.029 + 0.36 \times (RH/100)^{3.7}\right]}{0.029} \quad (4)$$

Where $\gamma_{RH=0}$ is the uptake coefficient under the dry condition. In this work, we chose $\gamma_{RH=0}$ to be 6×10^{-5} referring to previous studies on the interaction between SO_2 and dust particles (listed in **Supplementary Table 2**).

Supplementary Table 2. The uptake coefficients of SO_2 onto dust in the literatures

Reaction surfaces	$\gamma_{RH}=0$	References
Saharan dust	6.4×10^{-5}	Adams et al. (2005)[32]
China Loess	3.0×10^{-5}	Usher et al. (2002)[33]
Mixture A	7.1×10^{-5}	Gao et al. (2006)[34]
Mixture B	9.4×10^{-5}	Gao et al. (2006)[34]
Building dust	6.3×10^{-5}	Gao et al. (2006)[34]
Dust	4.0×10^{-5}	Crowley et al. (2010)[35]

3. Model evaluation

Meteorological parameters. The observation data from the National Climatic Data Center (NCDC) was used to evaluate the reliability of the meteorological prediction. The statistical performance of 10-m wind speed (WS10), 10-m wind direction (WD10), 2-m temperature (T2) and 2-m humidity (H2) for each month was listed in **Supplementary Table 3**. The statistical parameters contain bias, gross error (GE), root mean square error (RMSE), and the index of agreement (IOA), which are explained in detail in Baker et al. (2004)[36]. The values are generally within the benchmark range (suggested by Emery et al. (2001)[37]) and the model performance is reasonably acceptable.

Supplementary Table 3. Performance statistics of meteorological variables

			Jan	Feb	Mar	Apr	May	Jun	Jul	Aug	Sep	Oct	Nov	Dec	Benchmark
WS10	Bias	(m/s)	0.11	0.03	-0.02	-0.01	-0.04	-0.06	-0.07	-0.09	-0.05	0	0.09	0.09	⩽±0.5
	GE	(m/s)	1.1	1.14	1.17	1.19	1.12	1.1	1.09	1.09	1.07	1.06	1.11	1.07	⩽2
	RMSE	(m/s)	1.52	1.59	1.62	1.63	1.54	1.51	1.51	1.5	1.48	1.45	1.53	1.49	⩽2
	IOA		0.8	0.81	0.82	0.82	0.81	0.78	0.77	0.79	0.8	0.83	0.84	0.82	⩾0.6
WD10	Bias	(deg)	3.17	3.62	3.01	2.97	2. 73	2.5	1.86	-0.05	4.3	5.11	5.12	6.03	⩽±10
	GE	(deg)	44.08	41.23	40.43	40.32	41.53	42.29	42.04	46.88	47.27	46.26	44.88	46.89	⩽30
T2	Bias	(K)	-0.15	-0.4	-0.22	-0.19	-0.43	-0.24	-0.16	-0.14	0	0.12	0.05	0.09	⩽±0.5
	GE	(K)	2.17	2.07	2.12	2	2	1.86	1.73	1.73	1.67	1.75	1.89	2	⩽2
	RMSE	(K)	2.93	2.87	2.88	2.75	2.74	2.58	2.41	2.42	2.27	2.38	2.65	2.75	
	IOA		0.99	0.99	0.98	0.97	0.96	0.95	0.96	0.96	0.97	0.98	0.98	0.98	⩾0.8
H2	Bias	(g/kg)	0.02	0.12	0.02	-0.03	-0.5	-0.67	-0.83	-0.68	-0.38	-0.24	-0.19	-0.08	⩽±1
	GE	(g/kg)	0.69	0.74	0.95	1.13	1.6	1.83	2.01	1.92	1.48	1.15	0.85	0.67	⩽2
	RMSE	(g/kg)	1.04	1.13	1.39	1.6	2.24	2.52	2.93	2.76	2.1	1.66	1.28	1	
	IOA		0.98	0.98	0.97	0.97	0.96	0.94	0.92	0.94	0.96	0.96	0.97	0.97	⩾0.6

$PM_{2.5}$ concentration. The $PM_{2.5}$ concentrations for 74 monitoring cities in Mainland China obtained from the Ministry Environmental Protection of the People's Republic of China (**Supplementary Fig. 1**) were used to evaluate the model performance. As shown in **Supplementary Table 4**, the Normalized Mean Bias (NMBs) of seasonal $PM_{2.5}$ concentrations for winter, spring, summer and autumn are -9.9%, -13.2%, -4.6% and -2.8%, respectively. The correlation coefficients (R) are 0.72, 0.56, 0.63 and 0.73, respectively. It can be seen that the $PM_{2.5}$ concentrations are slightly underestimated for all the four seasons, which may be mainly attributed to the underestimation of secondary organic aerosols (SOA) and the exclusion of fugitive dust emissions from cropland. The daily averages of simulated and observed concentrations of $PM_{2.5}$ at three important cities are presented in **Supplementary Fig. 2**, including Beijing, Shanghai and Guangzhou. The NMBs of $PM_{2.5}$ predictions are 19.5%, -29% and -17%, respectively, and the correlation coefficients (R) are all above 0.5. Bias still existed for the $PM_{2.5}$ concentration predictions for some reasons. First, large uncertainties show in the estimation of fugitive dust emission because of limited local measurements for emission factors and the lack of accurate location information. In addition, the model system used in this study didn't include the aerosol direct effects, which could lead to underestimating the $PM_{2.5}$ concentrations during severe haze periods. Nevertheless, these results demonstrate the model could capture the $PM_{2.5}$ variation reasonably well.

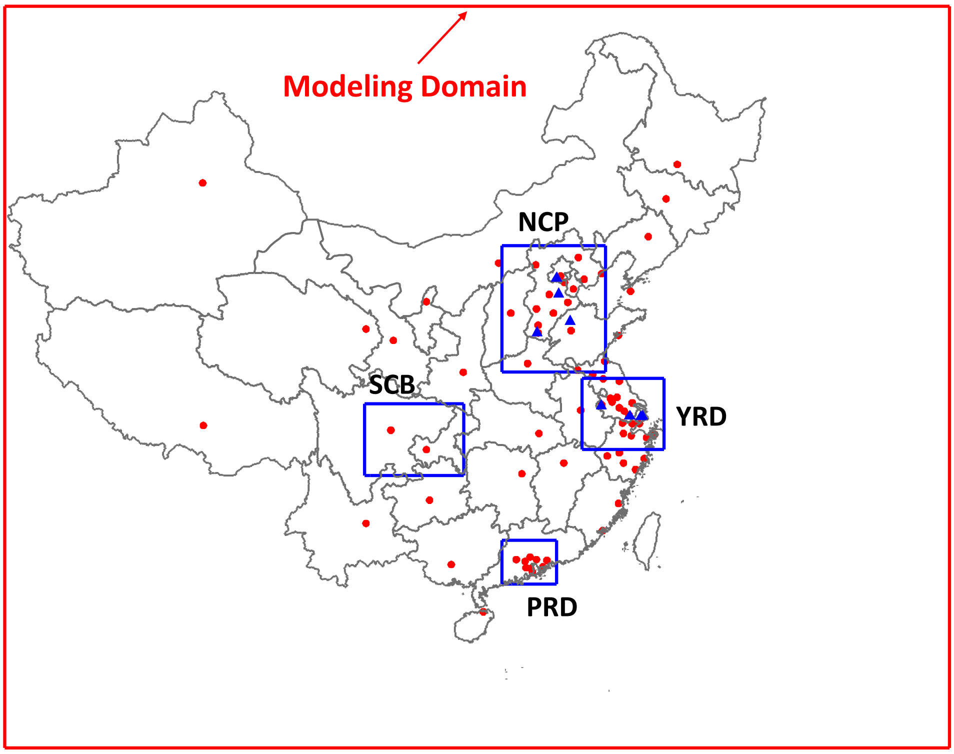

Supplementary Figure 1. The modeling domain (red rectangle), the four key regions (blue rectangles) and locations of observational data for model evaluation. The red circles represent 74 cities for $PM_{2.5}$ monitoring from MEP and the blue triangles represent 8 sites for SIA monitoring. This figure is produced using Arcgis, [version 9.3], (http://desktop.arcgis.com/) and Microsoft PowerPoint 2013 (https://www.microsoft.com/).

Supplementary Table 4. Model performance for seasonal $PM_{2.5}$ concentrations

	Mean Sim. (μg/m^3)	Mean Obs. (μg/m^3)	NMB	R
Winter	99.8	110.8	-9.9%	0.72
Spring	55.0	63.4	-13.2%	0.56
Summer	43.0	45.1	-4.6%	0.63
Autumn	65.2	67.1	-2.8%	0.73

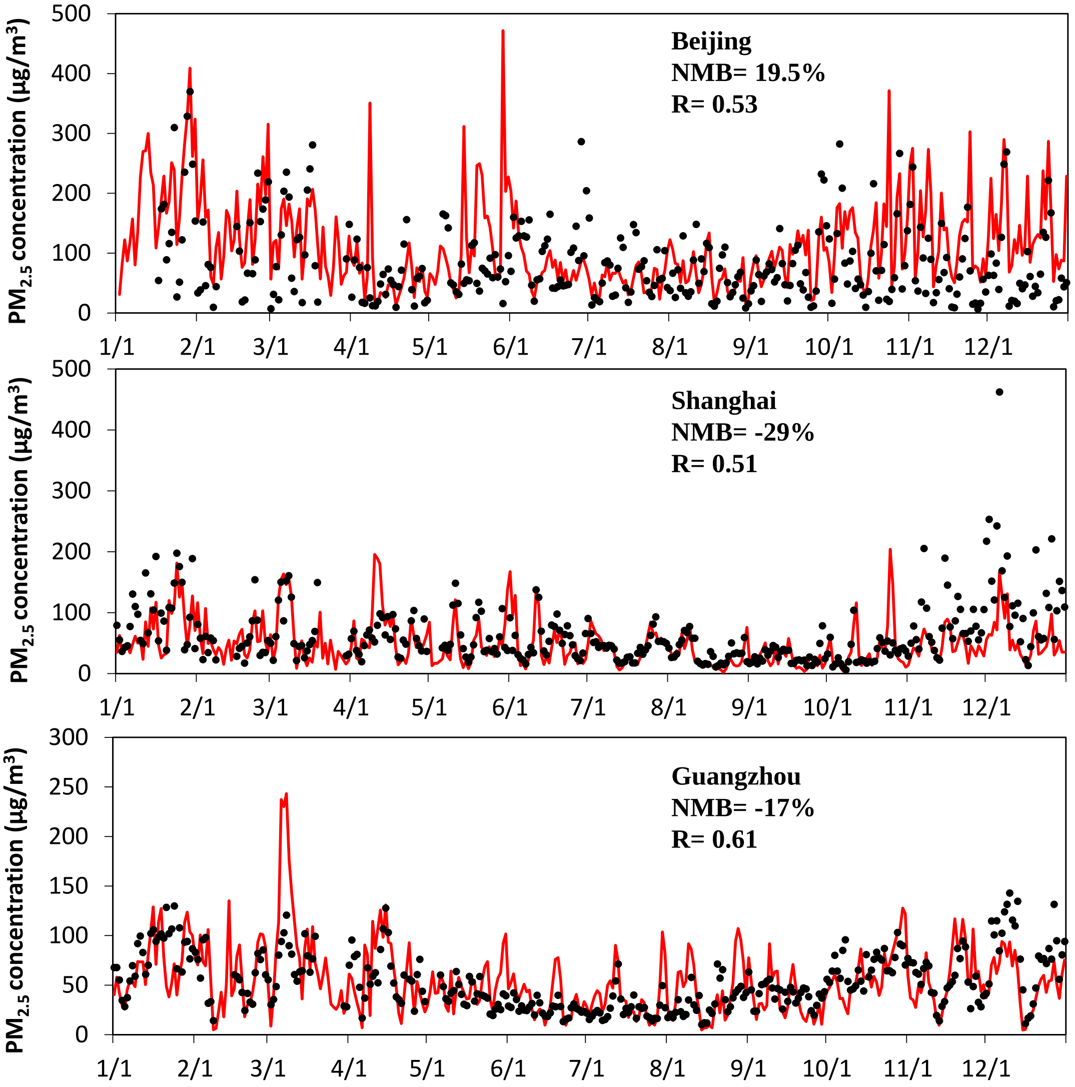

Supplementary Figure 2. Comparison of simulated daily $PM_{2.5}$ concentrations with observations at Beijing, Shanghai and Guangzhou.

Sulfate, nitrate and ammonium concentration. The observation data of SIA were very spare and not publicly accessible. In this study, the daily observations in eight monitoring sites were used to evaluate the model performance for SIA. The name and monitoring periods of each site are listed in **Supplementary Table 5**. **Supplementary Table 6** present the comparisons of the observations with the simulated results

Supplementary Table 5. The monitoring sites and periods for SIA modeling evaluation

Sites name	Monitoring periods	Data sources
Shanghai (2 sites), Suzhou, Nanjing	2011/6/1-2011/6/30<br>2011/7/20-2011/8/20<br>2011/10/20-2011/11/30<br>2011/12/20-2011/12/31	Tsinghua University
Handan	2013/1,2013/4,2013/7,2013/10	Hebei University of Engineering
Baoding, Dezhou	2013/7/21-2013/8/26<br>2013/11/20-2013/12/21	Peking University
Beijing	2013/11/25-2013/12/24	Tsinghua University

Supplementary Table 6. Model performance for daily SIA concentrations

		SO_4^{2-}	NO_3^-	NH_4^+
MeanObs		15.5	12	9.7
Simulation I	MeanSim	9.1	14.9	7.6
	NMB (%)	-41.3%	24.2%	-21.6%
	R	0.4	0.6	0.6
Simulation II	MeanSim	13.6	11.6	8.1
	NMB (%)	-12.3%	-3.3%	-16.5%
	R	0.5	0.6	0.6

4. Spatial and seasonal patterns of SIA over China.

Supplementary Figure 3 presents the spatial and seasonal distributions of SO_4^{2-}, NO_3^- and NH_4^+ over China.

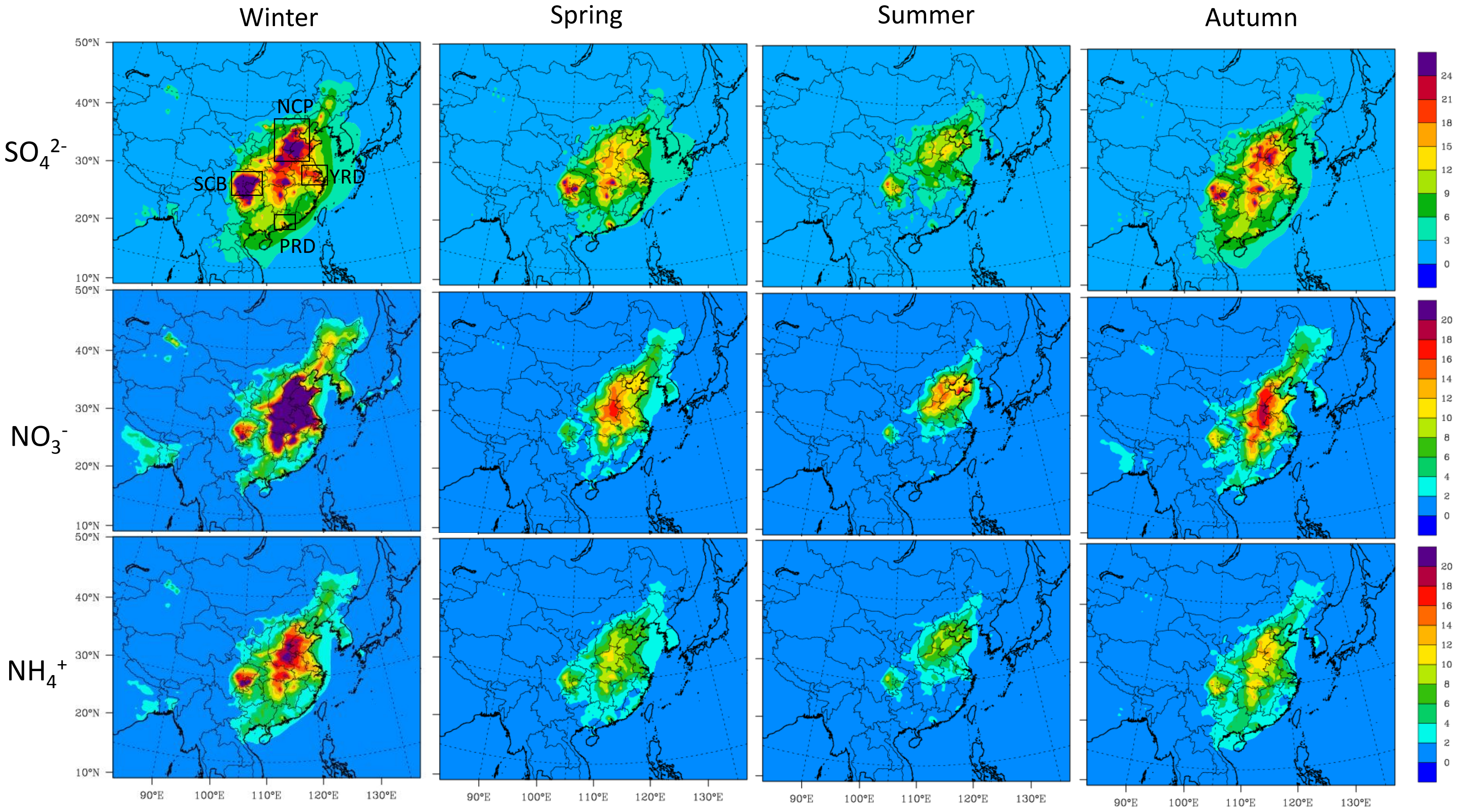

Supplementary Figure 3. Spatial distribution of simulated seasonal concentrations of sulfate, nitrate and ammonium over China in 2013. This figure is produced using the NCAR Command Language (Version 6.2.1) [Software]. (2014). Boulder, Colorado: UCAR/NCAR/CISL/TDD. http://dx.doi.org/10.5065/D6WD3XH5.

5. Sulfate enhancement by dust particles

As shown in **Supplementary Fig. 4**, an average sulfate concentration of 51.6μg/m^3 for the whole episode was measured at a site in Tsinghua University, Beijing (40.0°N, 116.3°E)[38]. The corresponding simulated average sulfate concentration for the whole episode increased from 18.4 to 30.6μg/m^3. The daily enhancement for sulfate concentration was highest in January 12, which was about 31μg/m^3.

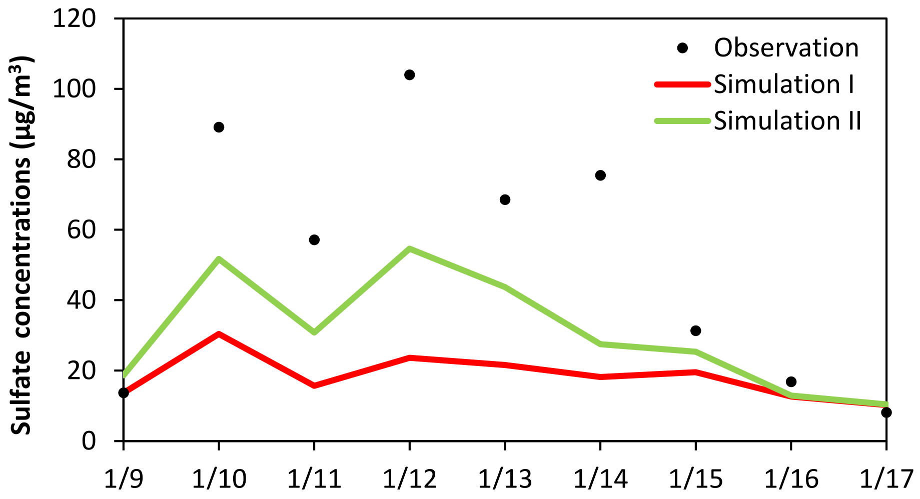

Supplementary Figure 4. The comparison of observed and simulated SIA concentrations from Simulation I and II. In Simulation I, the default CMAQ model was used. In Simulation II, the sulfate enhancement by dust was taken into consideration.

References

1 Fu, X. *et al.* Source, transport and impacts of a heavy dust event in the Yangtze River Delta, China, in 2011. *Atmospheric Chemistry and Physics* **14**, 1239-1254, doi:10.5194/acp-14-1239-2014 (2014).

2 Zheng, B. *et al.* High-resolution mapping of vehicle emissions in China in 2008. *Atmospheric Chemistry and Physics* **14**, 9787-9805, doi:10.5194/acp-14-9787-2014 (2014).

3 Liu, Z., Zhang, M., Hao, C. & Du, Y. Road Dust Emission Factors in Jinan City. *Environmental Science and Technology* **35**, 150-154 (2012).

4 Peng, K. *et al.* Emission factor and inventory of paved road fugitive dust sources in the Pearl River Delta region. *Acta Scientiae Circumstantiae* **33**, 2657-2663 (2013).

5 Xu, Y. & Zhou, Q. Emission characteristics and spatial distribution of road fugitive dust in Tianjin, China. *China Environmental Science* **32**, 2168-2173 (2012).

6 Huang, Y. *Reseach on estimation and distribution character of urban fugitive dust* Master thesis, East China Normal University, (2006).

7 Huang, Y. *et al.* Emission Factor and Size Dist ribution of Fugitive Dust from Construction Sites in Hohhot. *Journal of Inner Mongolia University (Natural Science Edition)* **42**, 230-235 (2011).

8 Tong, X., Qiao, Y., Yao, S. & Zhang, J. Emission Inventory of Construction Fugitive Dust in Nanjing. *Environmental monitoring management and technology* **26**, 21-24 (2014).

9 Wang, S., Wang, T., Shi, R. & Tian, J. Estimation of different fugitive dust emission inventory in Nanjing. *Journal of University of Chinese Academy of Sciences* **31**, 351-359 (2014).

10 Yang, Y. *Character, level and regulatory measures study of fugitive dust emissions from building construction sites in PRD* Master thesis, South China University of Technology, (2014).

11 Bao, Z., Feng, Y., Jiao, L., Hong, S. & Liu, W. Characterization and Source Apportionment of PM 2.5 and PM 10 in Hangzhou. *Environmental Monitoring in China* **26**, 44-48 (2010).

12 Ho, K. F., Lee, S. C., Chow, J. C. & Watson, J. G. Characterization of PM10 and PM2.5 source profiles for fugitive dust in Hong Kong. *Atmospheric Environment* **37**, 1023-1032, doi:10.1016/s1352-2310(02)01028-2 (2003).

13 Hua, L. *et al.* Analysis of PM10 Source Profiles in Beijing. *Environmental Monitoring in China* **22**, 64-71 (2006).

14 Ma, Z. *et al.* Study on the PM2.5 profiles of typical source in Beijing. *Acta Scientiae Circumstantiae* (2015).

15 Zhang, R. *et al.* Elemental profiles and signatures of fugitive dusts from Chinese deserts. *Science of the Total Environment* **472**, 1121-1129, doi:10.1016/j.scitotenv.2013.11.011 (2014).

16 Zhao, P. *Study on the assessment and control technology of urban dust pollution from construction and pavement* Doctor thesis, Nankai University, (2008).

17 Zheng, A., Yang, B., Wu, S., Wang, X. & Chen, X. Road Dust Loading and Chemical Composition at Major Cities in Fujian Province. *Environmental Science* **34**, 1901-1907 (2013).

18 Chen, J., Wang, W., Liu, H. & Ren, L. Determination of road dust loadings and chemical characteristics using resuspension. *Environmental Monitoring and Assessment* **184**, 1693-1709, doi:10.1007/s10661-011-2071-1 (2012).

19 Han, L., Zhuang, G., Cheng, S. & Wang, H. Characteristics of Resuspended Road Dust and Its Significant Effect on the Airborne Particulate Pollution in Beijing. *ENVIRONMENTAL SCIENCE* **30**, 1-8 (2009).

20 Huang, K. *The transformation of aerosol components during the long-range transport of Asian dust and the formation mechanism of haze in mega-city, China* Doctor thesis, Fudan University, (2010).

21 Wang, Q. *The mixing and interaction of Asian dust with the typical atmospheric pollutants during the long-range transport and its impact on urban air quality* Doctor thesis, Fudan University, (2012).

22 Alexander, B., Park, R. J., Jacob, D. J. & Gong, S. Transition metal-catalyzed oxidation of atmospheric sulfur: Global implications for the sulfur budget. *Journal of Geophysical Research-Atmospheres* **114**, doi:10.1029/2008jd010486 (2009).

23 Huang, X. *et al.* Pathways of sulfate enhancement by natural and anthropogenic mineral aerosols in China. *Journal of Geophysical Research-Atmospheres* **119**, 14165-14179, doi:10.1002/2014jd022301 (2014).

24 Siefert, R. L., Johansen, A. M., Hoffmann, M. R. & Pehkonen, S. O. Measurements of trace metal (Fe, Cu, Mn, Cr) oxidation states in fog and stratus clouds. *Journal of the Air & Waste Management Association* **48**, 128-143 (1998).

25 Zhao, B. *et al.* Impact of national NOx and SO2 control policies on particulate matter pollution in China. *Atmospheric Environment* **77**, 453-463, doi:10.1016/j.atmosenv.2013.05.012 (2013).

26 Fu, X. *et al.* Estimating NH3 emissions from agricultural fertilizer application in China using the bi-directional CMAQ model coupled to an agro-ecosystem model. *Atmospheric Chemistry and Physics* **15**, 6637-6649, doi:10.5194/acp-15-6637-2015 (2015).

27 Guenther, A. *et al.* Estimates of global terrestrial isoprene emissions using MEGAN (Model of Emissions of Gases and Aerosols from Nature). *Atmospheric Chemistry and Physics* **6**, 3181-3210 (2006).

28 Wang, K., Zhang, Y., Nenes, A. & Fountoukis, C. Implementation of dust emission and chemistry into the Community Multiscale Air Quality modeling system and initial application to an Asian dust storm episode. *Atmospheric Chemistry and Physics* **12**, 10209-10237, doi:10.5194/acp-12-10209-2012 (2012).

29 Li, J., Shang, J. & Zhu, T. Heterogeneous reactions of SO2 on ZnO particle surfaces. *Sci. China-Chem.* **54**, 161-166, doi:10.1007/s11426-010-4167-9 (2011).

30 Shang, J., Li, J. & Zhu, T. Heterogeneous reaction of SO2 on TiO2 particles. *Sci. China-Chem.* **53**, 2637-2643, doi:10.1007/s11426-010-4160-3 (2010).

31 Sun, Y. *et al.* The impact of relative humidity on aerosol composition and evolution processes during wintertime in Beijing, China. *Atmospheric Environment* **77**, 927-934, doi:10.1016/j.atmosenv.2013.06.019 (2013).

32 Adams, J. W., Rodriguez, D. & Cox, R. A. The uptake of SO2 on Saharan dust: a flow tube study. *Atmospheric Chemistry and Physics* **5**, 2679-2689 (2005).

33 Usher, C. R., Al-Hosney, H., Carlos-Cuellar, S. & Grassian, V. H. A laboratory study of the heterogeneous uptake and oxidation of sulfur dioxide on mineral dust particles. *Journal of Geophysical Research-Atmospheres* **107**, doi:471310.1029/2002jd002051 (2002).

34 Gao, Y. C. & Chen, D. Heterogeneous reactions of sulfur dioxide on dust. *Science in China Series B-Chemistry* **49**, 273-280, doi:10.1007/s11426-006-0273-0 (2006).
35 Crowley, J. N. *et al.* Evaluated kinetic and photochemical data for atmospheric chemistry: Volume V - heterogeneous reactions on solid substrates. *Atmospheric Chemistry and Physics* **10**, 9059-9223, doi:10.5194/acp-10-9059-2010 (2010).
36 Baker, K., Johnson, M. & King, S. Meteorological Modeling Protocol for Application to PM2.5/haze/ ozone Modeling Projects. (Lake Michigan Air Directors Consortium, Des Plaines, Illinois, 2004).
37 Emery, C., Tai, E. & Yarwood, G. *Enhanced meteorological modeling and performance evaluation for two texas episodes. Report to the Texas Natural Resources Conservation Commission.*, (2001).
38 Cao, C. *et al.* Inhalable Microorganisms in Beijing's PM2.5 and PM10 Pollutants during a Severe Smog Event. *Environ. Sci. Technol.* **48**, 1499-1507, doi:10.1021/es4048472 (2014).
